# Seizures After Lumbar Laminectomy: A Clinical Case

**DOI:** 10.7759/cureus.100516

**Published:** 2025-12-31

**Authors:** Neuza Machado, Elisabete Monteiro, Juliana Branquinho, Mafalda Neves, Sofia Mendes

**Affiliations:** 1 Anesthesiology, Unidade Local de Saúde Viseu Dão-Lafões, Viseu, PRT; 2 Intensive Care Medicine, Unidade Local de Saúde de São João, Porto, PRT; 3 Surgery and Physiology, Faculty of Medicine, University of Porto, Porto, PRT; 4 Intensive Care Medicine, Unidade Local de Saúde de Castelo Branco, Castelo Branco, PRT

**Keywords:** cerebrospinal fluid hypotension, incidental durotomy, lumbar decompression, neurointensive care, seizures

## Abstract

Incidental durotomy can occur as a complication of spine surgery, potentially resulting in serious intracranial complications. We report a case of a 71-year-old female with significant spinal stenosis from L3-L5 who underwent a posterior lumbar decompression and fusion. In the immediate post-operative period, she developed generalized tonic-clonic seizures. A brain computed tomography scan was urgently performed, showing a slight decrease in the amplitude of the supratentorial ventricular system, associated with engorgement of the venous sinuses, interpeduncular hyperdensity, and thalamic hypodensities (evoking a pseudohypoxic pattern). These changes, taken together, were consistent with the suspicion of cerebrospinal fluid (CSF) hypotension. A brain magnetic resonance image was done, confirming a pseudohypoxic cerebral pattern. This case illustrates CSF hypotension after iatrogenic dural tear, manifested by generalized seizures. Repairing an incidental durotomy should be done as early as possible to reduce CSF leakage and prevent the devastating effects of CSF hypotension.

## Introduction

Spine surgery carries the risk of various complications, such as damage to blood vessels, injury to nerve roots, formation of a hematoma in the epidural space after surgery, infections at the surgical site, spinal cord damage, and tears in the dura mater [1]. Unintentional durotomy with cerebrospinal fluid (CSF) leakage has also been observed following procedures such as lumbar puncture, spinal anesthesia, myelography, and the placement of a lumbar drain [2,3]. Various treatment approaches are available for managing durotomies, including direct primary closure during surgery and the use of fibrin glue, fat grafts, or muscle grafts to aid in sealing the tear [4]. The precise pathophysiology remains unclear, but it is believed that a CSF leak can lead to reduced intracranial pressure and enlargement of the subdural spaces. This may result in downward displacement of the brain, causing stretching of neural structures [5]. We present a case of CSF hypotension following an incidental dural tear during posterior lumbar spinal surgery.

## Case presentation

**Figure 1:**
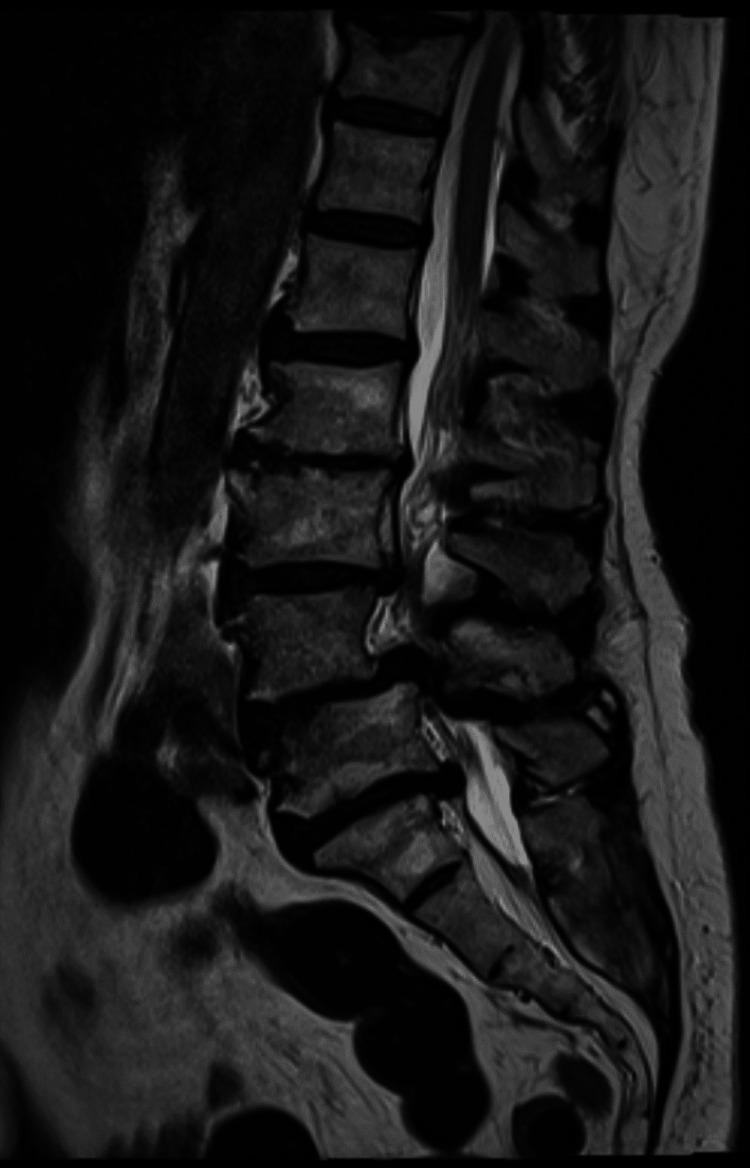
Pre-operative lumbar magnetic resonance imaging

A 71-year-old female presented with lumbar pain, numbness and weakness of both legs. Her past medical history was only remarkable for type 2 diabetes mellitus. No history of hypertension or coagulation disorders was documented. Pre-operative imaging lumbar spine scan showed significant stenosis at the level of L3-L5 (Figure 1). Laboratory blood tests were all within normal limits.

**Figure 2:**
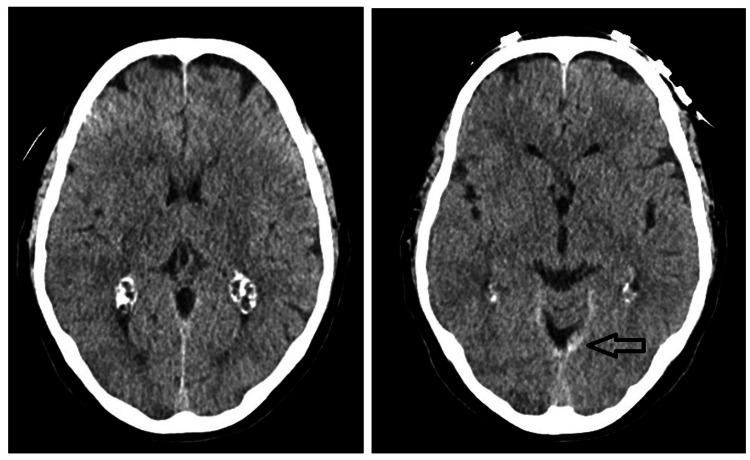
Post-operative brain computed tomography There is a slight decrease in the amplitude of the supratentorial ventricular system, associated with engorgement of the venous sinuses, interpeduncular hyperdensity, and hypodensity in the thalami (evoking a pseudohypoxic pattern). There is also the appearance of "pseudo-subarachnoid hemorrhage" (arrow).

She underwent a posterior lumbar decompression and fusion L3-L5. The anesthetic technique was total intravenous anesthesia. The intra-operative analgesia was paracetamol 1g, metamizole 1g, ketorolac 30mg and morphine 2mg. The procedure was performed in a prone position. The neurosurgeons did not see any signs of intraoperative CSF leak. The surgery was completed and the patient was awoken and extubated. After a few minutes, generalized tonic-clonic seizures occurred. As a result, she was promptly re-intubated and transferred to the Neurocritical Care Unit (NCCU). An urgent brain computed tomography (CT) scan was ordered. It showed a decrease in the amplitude of the supratentorial ventricular system, associated with engorgement of the venous sinuses, interpeduncular hyperdensity and hypodensity in the thalami (evoking a pseudohypoxic pattern) (Figure 2).

Different aetiologies were considered, including a dural tear with CSF leak or an anaesthetic complication.

Seizures were managed with sedation (propofol) and antiepileptics (levetiracetam). An electroencephalogram (EEG), performed without sedation, revealed diffuse slowing of brain electrical activity, consistent with moderate encephalopathy, and no apparent epileptic activity.

**Figure 3:**
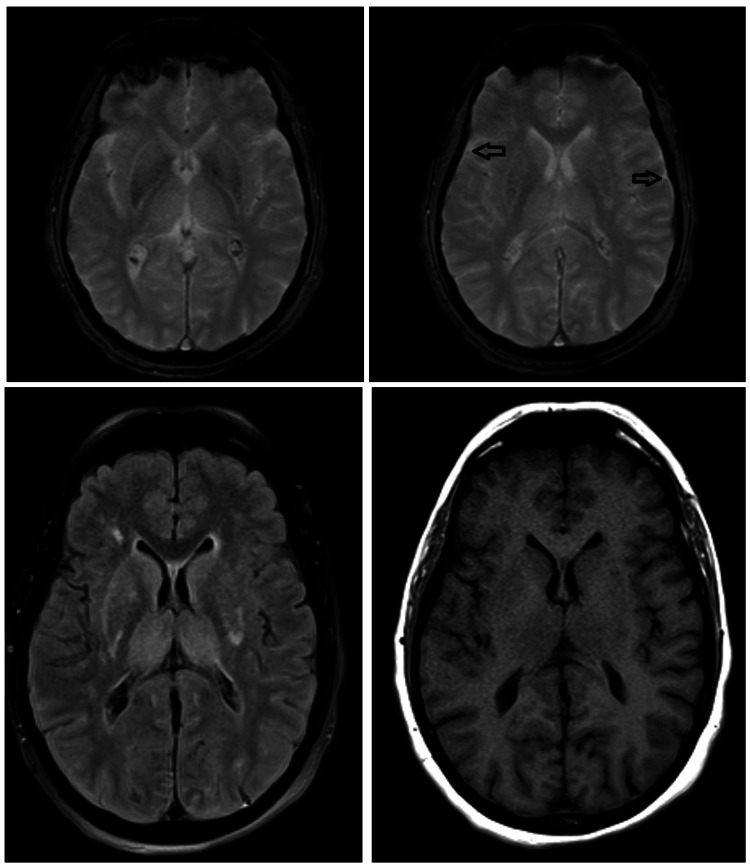
Post-operative brain magnetic resonance imaging Changes consistent with a pseudohypoxic pattern are described in the context of spinal surgical intervention. There was a diffuse thickening and pachymeningeal enhancement (arrows); these findings are compatible with a context of cerebrospinal fluid hypotension.

A wake-up test showed a persistent Glasgow Coma Scale (GCS) of 3 (E1V1M1) [6], and a brain magnetic resonance imaging (MRI) revealed a pseudohypoxic cerebral pattern (Figure 3).

**Figure 4:**
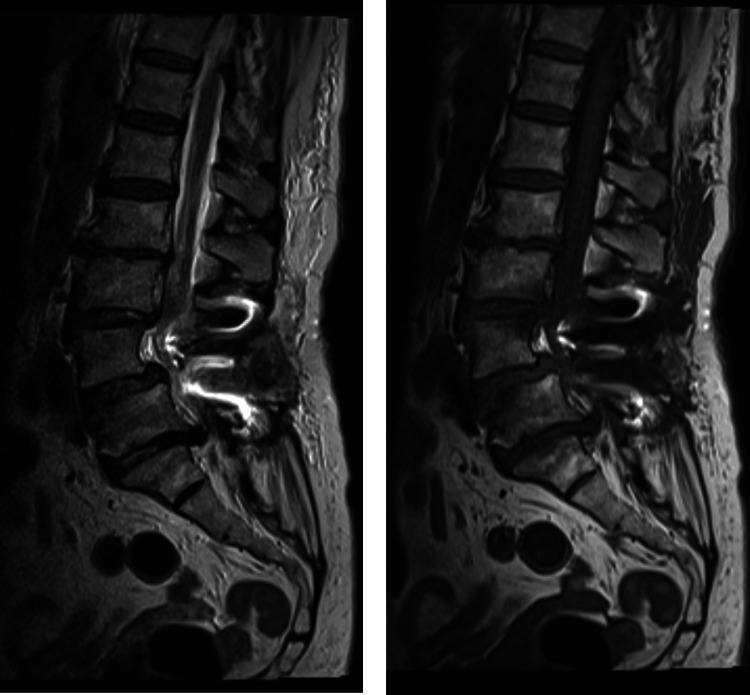
Post-operative lumbar images The MRI was unable to document liquor leak points.

A lumbar MRI was not able to document liquor leak points (Figure 4).

Intracranial pressure (ICP) was sustained at 2-4 mmHg with the head up to 30º. After several observations, on the sixth day of hospitalization, clear fluid drainages were observed through the lumbar surgical wound. With these drainages, ICP dropped to negative values. She initiated meropenem and vancomycin due to suspected central nervous system infection.

In a multidisciplinary discussion between neurosurgeons and neurointensivists, it was decided to perform a surgical review of the wound. A surgical exploration was performed in the operating room, with identification of one dural laceration and exteriorization of the rootlets adjacent to the right L5 screw. This dural tear was repaired with a dural substitute and biological glue.

On the twelfth day of hospitalization, four days after the second surgery, she was extubated and she presented with a GCS of 14 (E4V4M6) [6]. Her wound healed without signs of infection. A follow-up brain CT scan postoperatively demonstrated no alterations. At NCCU discharge, she was GCS 15 (E4V5M6). Her neurological examination showed improvement of her bilateral leg numbness compared to her pre-operative status, but mild cognitive and motor impairment (global strength 3+) was noted. She underwent physiotherapy and rehabilitation with posterior improvement (global strength 4+).

## Discussion

Dural injuries are a relatively uncommon complication of spine surgery, occurring in approximately 4% to 9% of cases [7]. Several causes of CSF leakage due to dural tears during surgery have been identified, including direct trauma to the dura, excessive traction on nerve roots, and improperly placed surgical instruments [8]. Risk factors that may contribute to this complication include advanced age, female sex, previous surgeries leading to scar tissue formation, corrective vertebral osteotomy procedures, degenerative spondylolisthesis, and earlier radiotherapy, which can hinder proper tissue healing [9,10]. In the majority of cases, patients experience a persistent headache caused by the downward shift of the brain, which places tension on the pain-sensitive dural sinuses [11]. In our case, the patient did not present with any preceding symptoms. Instead, she presented with a new-onset, sudden generalized tonic-clonic seizure that occurred immediately after the surgery. Our patient was healthy and had no history of hypertension or previous head, thoracic or lumbar trauma.

Although the exact mechanism is not fully understood, it is believed that a continuous loss of CSF leads to a sustained decrease in ICP. This reduction in pressure causes the brain, which typically floats in CSF, to descend or sag within the skull. As a result, sensitive structures such as the meninges and bridging veins are stretched, which is thought to be the source of symptoms like headache and discomfort [12]. Some researchers also propose that a decrease in CSF pressure can lead to downward displacement of the brain. Under normal conditions, CSF provides buoyancy that helps support the brain’s weight. When CSF pressure drops, this support is reduced, allowing the brain to sag or shift downward, which may result in a range of neurological symptoms. We believe that the cause of our patient was the sudden loss of a significant volume of CSF through the dural tear.

A brain CT scan is essential for diagnosis and should be conducted promptly after the onset of any worrisome symptoms [13]. The scan may reveal downward displacement of the brain and herniation of the cerebellar tonsils, reduced ventricular size, enhancement of the meninges, or enlarged epidural veins [13]. Intracranial hypotension is challenging to confirm definitively using axial head CT scans. Common findings may include narrowing of the basal cisterns that seems disproportionate to any extra-axial fluid collections, collapse of the third ventricle, and widespread brain swelling. However, CT scans rarely provide a conclusive diagnosis. As a result, diagnosing intracranial hypotension relies largely on the patient's clinical history, with additional imaging performed when there is strong clinical suspicion [14]. In our patient, the brain CT scan demonstrated a decrease in the amplitude of the supratentorial ventricular system, associated with engorgement of the venous sinuses, interpeduncular hyperdensity and hypodensity in the thalami (evoking a pseudohypoxic pattern).

Management of a CSF leak can involve conservative, multifaceted approaches such as bed rest, adequate hydration, and pain relief, particularly in cases of mild CSF hypotension without severe symptoms. However, surgical treatment is often necessary for patients experiencing significant CSF loss accompanied by severe neurological deficits [15]. Most reported cases generally follow a benign course and result in good neurological recovery. Although the overall incidence is relatively low, approximately 5 in 100000, it is believed that many cases go undiagnosed [16]. Intracranial hypotension leading to reversible coma has a low mortality rate, with some studies reporting death rates below 10% [17]. Although coma in this situation is a serious complication, it is frequently reversible when treated promptly and appropriately. In the present case, our patient was managed in the NCCU with surgical intervention. At discharge, she was GCS 15 (E4V5M6) [6] and her neurological examination showed mild cognitive and motor impairment (global strength 3+), so she was orientated to physiotherapy and rehabilitation.

## Conclusions

CSF hypotension is an uncommon but potentially serious complication that can occur following a CSF leak caused by iatrogenic durotomy during spinal surgery. The appearance of seizures after spine surgery should raise suspicion for postoperative CSF hypotension.

Prompt surgical repair of an incidental durotomy is crucial to minimize CSF leakage. Additionally, a brain CT scan should be performed to confirm the diagnosis and rule out other possible structural causes.
